# A Competing Risk Analysis Model to Determine the Prognostic Value of Isolated Tumor Cells in Axillary Lymph Nodes for T1N0M0 Breast Cancer Patients Based on the Surveillance, Epidemiology, and End Results Database

**DOI:** 10.3389/fonc.2020.572316

**Published:** 2020-09-18

**Authors:** Yijun Li, Huimin Zhang, Wei Zhang, Yu Ren, Yan Qiao, Kunlong Li, Heyan Chen, Shengyu Pu, Jianjun He, Can Zhou

**Affiliations:** ^1^Department of Breast Surgery, First Affiliated Hospital of Xi’an Jiaotong University, Xi’an, China; ^2^School of Medicine, Xi’an Jiaotong University, Xi’an, China

**Keywords:** competing risk model, SEER database, female breast cancer, survival analysis, isolated tumor cells

## Abstract

**Introduction:**

Knowledge of the association between isolated tumor cells (ITCs) in breast cancer patients and the outcome is very limited. We aimed to determine the prognostic value of axillary lymph node ITCs for T1N0M0 female breast cancer (FBC) patients.

**Methods:**

Data for T1N0M0 FBC patients staged ITCs negative [pN0(i−)] and positive [pN0(i+)] were extracted from the Surveillance, Epidemiology, and End Results database from 2004 to 2015. Prognostic predictors were identified by Kaplan–Meier analysis, competing risk model, and Fine–Gray multivariable regression model.

**Results:**

A total of 94,599 subjects were included, 88,632 of whom were staged at pN0(i−) and 5,967 were pN0(i+). Patients staged pN0(i+) had worse breast cancer-specific survival (BCSS) [hazard ratio (HR): 1.298, 95% CI = 1.069–1.576, *P* = 0.003] and higher breast cancer-specific death (BCSD) rate (Gray’s test, *P* = 0.002) than pN0(i−) group. In the Fine–Gray multivariable regression analysis, the pN0(i+) group had higher BCSD rate (HR: 1.321, 95% CI = 1.109–1.575, *P* = 0.002) than pN0(i−) group. In subgroup analyses, no significant difference in BCSD was shown between the chemotherapy and non-chemotherapy subgroup (Gray’s test, *P* = 0.069) or radiotherapy and non-radiotherapy subgroup (Gray’s test, *P* = 0.096).

**Conclusion:**

ITC was independently related to the increase of the BCSD rate and could be identified as a reliable survival predictor for T1N0M0 FBC patients.

## Introduction

Isolated tumor cells (ITCs), proposed by Saphir and Amromin (1) as occult metastasis of axillary lymph nodes in breast cancer, were defined as the single tumor cell or tumor-cell cluster with a maximum diameter of no more than 0.2 mm. In 2002, the 6th edition of the American Joint Committee on Cancer (AJCC) TNM classification manual (2)included the existence of ITC in its lymph node staging system and proposed the definition of pN0(i**−**) and pN0(i+). N is staged at pN0 if there is no evidence of the tumor in the lymph node by hematoxylin and eosin (H&E) staining. If neither H&E nor immunohistochemical (IHC) staining detects the presence of tumor cells in the lymph node, the N stage is classified as pN0(i**−**). If ITC is found in lymph node according to H&E or IHC staining, the N stage is defined as pN0(i+).

Due to the lack of large multicenter randomized controlled clinical study and long follow-up time, the impact of ITCs on patients’ survival is controversial. Some studies showed that ITCs had little effect on prognosis (3–9), while other researches suggested that ITCs had an adverse influence on survival (10–15). Therefore, more studies are urgently needed to confirm the real-world curative effect of lymph node ITCs in patients with female breast cancer (FBC).

To further explore and identify the prognostic value of axillary lymph nodes ITCs in patients with T1N0M0 FBC, we followed up a large cohort of FBC patients from 2004 to 2015 by using the population-based Surveillance, Epidemiology, and End Results (SEER) database. Statistical methods such as Kaplan–Meier analysis and competing risk model were applied to investigate the effect of axillary lymph node ITCs on the prognosis of T1N0M0 FBC.

## Materials and Methods

### Data Resource

The SEER database, maintained by the National Cancer Institute, is the world’s largest public cancer dataset. The SEER program consists of 18 cancer registries and collects the demography, clinical characteristics, and survival information of cancer in representative geographic regions of the United States, which covers approximately 26% of the United States population (16). The relevant data were extracted from the SEER^∗^Stat software version 8.3.6^1^ (Information Management Service, Inc., Calverton, MD, United States). All procedures were performed in accordance with approved guidelines. Because the SEER database is publicly accessible, this study does not require informed patient consent and was deemed exempt from review by the Ethics Committee of the First Affiliated Hospital of Xi’an Jiaotong University.

### Patient Cohort

Patients diagnosed with breast cancer from 2004 to 2015 were enrolled in the study, and we adopted the AJCC staging system (6th Edition) to define tumor stage. Patients were included if they met the following criteria: (1) female; (2) primary breast cancer (ICD-0-3 histology codes: 8430/3, 8440/3, 8453/3, 8460/3, 8460/3, 8470/3, 8480/3, 8500/3, 8501/3, 8502/3, 8503/3, 8504/3, 8507/3, 8510/2, 8513/3, 8514/3, 8520/3, 8521/3, 8522/3, 8523/3, 8524/3, 8525/3, 8530/3, 8540/3, 8541/3, 8542/3, 8543/3, 8560/3, 8570/3, 8571/3, 8572/3, 8573/3, 8574/3, 8575/3); (3) aged 18 years or older; (4) T1 and M0 stages; and (5) axillary lymph node status of pN0(i+) or pN0 (i**−**). The following demographic and clinicopathological variables were included: age at diagnosis, race, laterality, grade of tumor, T stage, N stage, M stage, estrogen receptor (ER) phenotype, progesterone receptor (PR) phenotype, surgery status, radiotherapy status, chemotherapy status, survival months, vital status, cause of death, and marital status.

**FIGURE 1:**
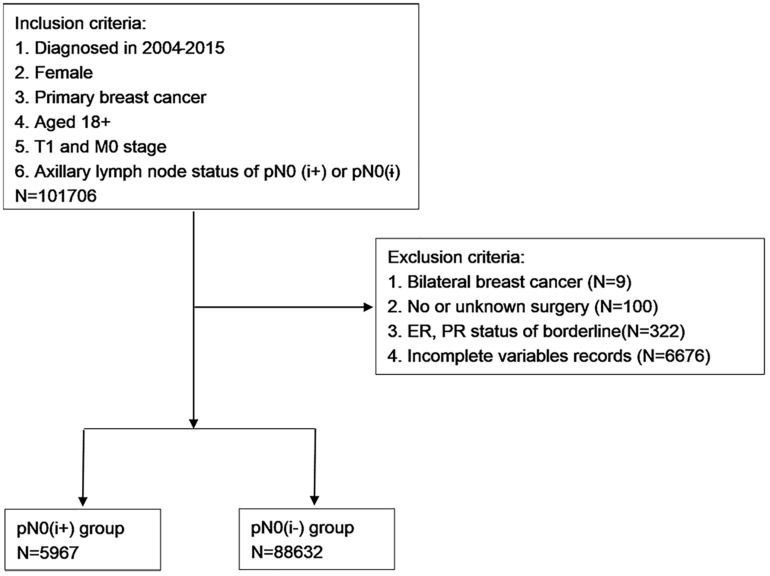
Eligibility, inclusion, and exclusion criteria of the study population.

After the preliminary selection, patients were excluded by the following criteria: (1) bilateral breast cancer; (2) no or unknown surgery; (3) ER and PR status of borderline; and (4) incomplete variables records. The selection procedure is shown in Figure 1.

A total of 94,599 patients with T1N0M0 FBC were selected. To evaluate the effect of lymph nodes ITCs on prognosis, the study cohort was divided into two groups by the status of ITCs: the pN0(i**−**) group and pN0(i+) group. “No radiation and/or cancer-directed surgery” was considered as no radiotherapy. “No/unknown” chemotherapy recodes were considered as no chemotherapy.

#### Endpoints

Patients were followed up until November 2018, and the median follow-up time was 5.75 years (ranging from 0 to 12.92 years). The primary endpoint was breast cancer-specific survival (BCSS), while the secondary was overall survival (OS) and breast cancer-specific death (BCSD). OS was referred to the time from the date of diagnosis to the date of death, the date of the last follow-up, or November 2018. BCSS and BCSD were measured as the time from the date of diagnosis to the date of death from breast cancer.

### Statistical Analysis

Descriptive statistics and frequency tables were used to summarize the baseline characteristics of patients. Categorical data were analyzed with the chi-squared test, and continuous data were compared with the Mann–Whitney U test. Kaplan–Meier curve analysis was employed to generate OS and BCSS curves, with the log-rank test was performed to determine the statistical differences among groups. The competing risk model analysis classified death causes into the BCSD group and non-BCSD group, and Gray’s test was used to identify statistical differences between BCSD and non-BCSD due to any competing risk events. The Fine–Gray multivariable regression model was performed to identify factors associated with the risk of death from all causes, which aimed to reduce bias caused by informative censoring. Two-sided *P*-values < 0.05 were used to determine statistical significance in all analyses. SPSS (version 22.0, IBM Corporation, Armonk, NY, United States) and R version 3.6.2 software (The R Foundation for Statistical Computing, Vienna, Austria^2^) were used to perform the calculations.

## Results

### Patient Characteristics

The baseline clinical characteristics of the included patients are shown in Table 1. Among the 94,599 patients, 88,632 (93.69%) were staged at pN0(i**−**), while 5,967 (6.31%) were staged at pN0(i+). The mean age at initial diagnosis was 60.79 (± 12.15) years. In total, 70,237 (74.25%) cases were non-Hispanic White, and 46,859 (49.53%) patients had tumors located in the left breast and 47,740 (50.47%) in the right breast. Moderately differentiated (grade II) tumors were most common in participants (31.42%). Of all patients, 82,246 (86.94%) were ER-positive, 73,121 (77.30%) were PR-positive, and 59,624 (63.03%) were married. A total of 68,404 (72.31%) patients were treated with breast-conserving surgery (BCS), 56,530 (59.76%) were treated with radiotherapy, and 21,584 (22.82%) were treated with chemotherapy. By comparing the pN0(i+) and pN0(i**−**) groups, significant differences (*P* < 0.05) were found in age, grade level, ER status, PR status, surgical methods, and radiotherapy and chemotherapy status. Patients with ITCs tend to be younger and had higher tumor grades. The pN0(i**−**) group had a lower positive rate of ER and PR. In terms of treatment, the pN0(i**−**) patients tend to receive BCS and radiotherapy, while the pN0(i+) patients were more likely to choose mastectomy and chemotherapy.

**TABLE 1:** The clinicopathological characteristics of female breast cancer patients with axillary lymph node status of pN0(i+) and pN0(i−).

Characteristics	N (94,599)		N0(i−) (88,632)		N0(i+) (5,967)		*P*
	N	%	n	%	n	%	
Age (years, mean ± SD)	60.79 ± 12.15	–	60.94 ± 12.13	–	58.48 ± 12.27	–	**<0.001**
Race							0.428
Non-Hispanic White	70,237	74.25	65,815	74.26	4,422	74.11	
Non-Hispanic Black	7,565	8.00	7,060	7.97	505	8.46	
Hispanic (all races)	8,906	9.41	8,342	9.41	564	9.45	
Other races	7,981	8.34	7,415	8.37	476	7.98	
Laterality							0.433
Left	46,859	49.53	43,874	49.50	2,985	50.03	
Right	47,740	50.47	44,758	50.50	2,982	49.97	
Grade							**<0.001**
1	29,724	31.42	28,232	31.85	1,492	25.00	
2	43,407	45.89	40,389	45.57	3,018	50.58	
3/4	21,468	22.69	20,011	22.58	1,457	24.42	
ER							**<0.001**
Positive	82,246	86.94	76,932	86.80	5,314	89.06	
Negative	12,353	13.06	11,700	13.20	653	10.94	
PR							**<0.001**
Positive	73,121	77.30	68,365	77.12	4,765	79.86	
Negative	21,478	22.70	20,276	22.88	1,202	20.14	
Marital status							0.596
Married	59,624	63.03	55,844	63.01	3,780	63.35	
Unmarried/DSW	34,975	36.97	32,788	36.99	2,187	36.65	
Surgery							**<0.001**
BCS	68,404	72.31	64,697	73.00	3,707	62.13	
Mastectomy	26,195	27.69	23,935	27.00	2,260	37.87	
Radiotherapy							**<0.001**
Yes	56,530	59.76	53,343	60.18	3,187	53.41	
No	38,069	40.24	35,289	39.82	2,780	46.59	
Chemotherapy							**<0.001**
Yes	21,584	22.82	19,703	22.23	1,881	31.52	
No	73,015	77.18	68,929	77.77	4,086	68.48	

*ER, estrogen receptor; PR, progesterone receptor; DSW, divorced and separated and widowed; BCS, breast-conserving surgery. Bold values means P < 0.05.*

### Kaplan–Meier Survival Analysis

**FIGURE 2:**
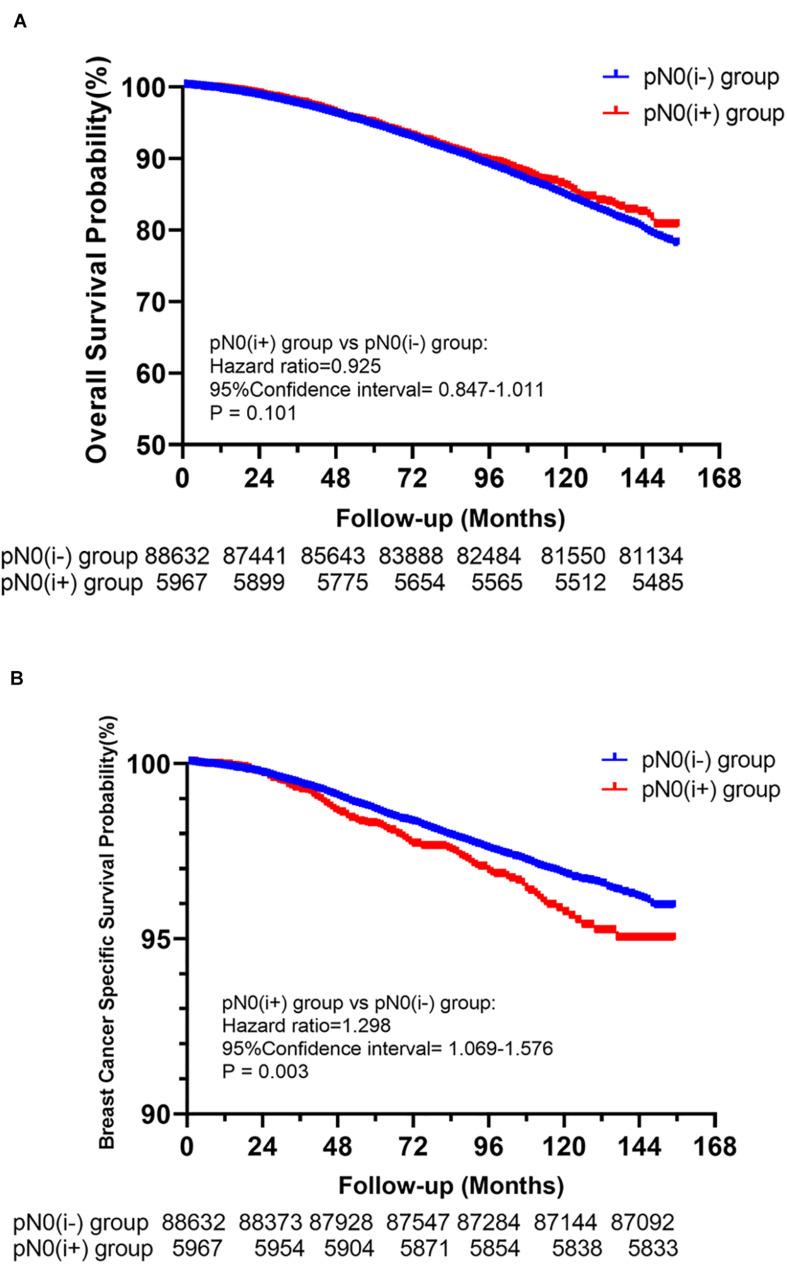
Kaplan–Meier survival analysis for pN0(i+) and pN0(i–) female breast cancer patients. **(A)** Overall survival curves for the pN0(i+) group and pN0(i–) group. **(B)** Breast cancer-specific survival curves for the pN0(i+) group and pN0(i–)group.

Among the 94,599 patients included, 8,034 (8.49%) died in this cohort study. The cumulative incidence of BCSD was only 1.78% (1,685/94,599), but the cumulative non-BCSD incidence was as high as 6.71% (6,349/94,599). Compared with patients in the pN0(i**−**) group, as shown in Figures 2A,B, patients in the pN0(i+) group had worse BCSS [hazard ratio (HR):1.298, 95% confidence interval (CI) = 1.069–1.576, *P* = 0.003]. There was no statistical difference between the pN0(i**−**) group and pN0(i+) group in OS (HR: 0.925, 95% CI = 0.847–1.011, *P* = 0.101).

### Competing Risk Model of Breast Cancer-Specific Death and Non-breast Cancer-Specific Death

**FIGURE 3:**
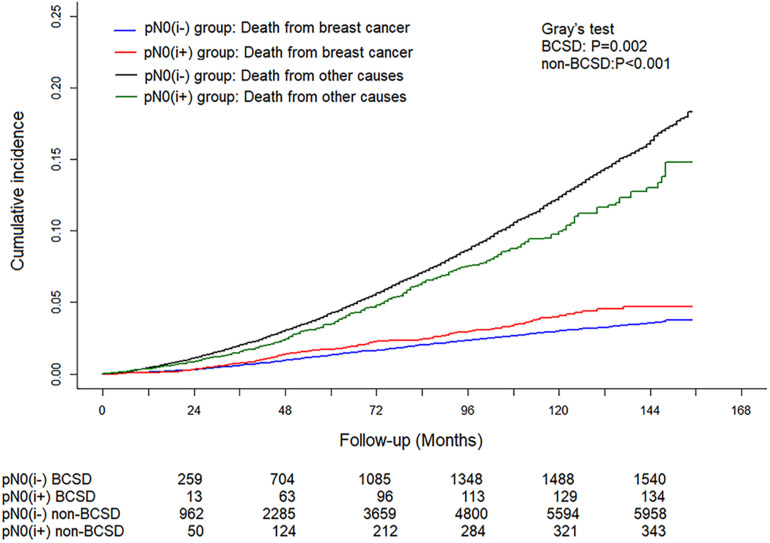
Cumulative incidence of breast cancer-specific deaths (BCSDs) and non-breast cancer-specific deaths (non-BCSDs) in pN0(i+) and pN0(i–) female breast cancer patients.

A total of 8,034 deaths were included in the matched cohort, of which 20.97% (1,685/8,034) were BCSD and 79.03% (6,349/8,034) were non-BCSD. As shown in Figure 3, patients in the pN0(i+) group had a higher cumulative BCSD rate (Gray’s test, *P* = 0.002) but lower non-BCSD rate (Gray’s test, *P* < 0.001) than patients in the pN0(i−) group.

### Multivariable Competing Risk Analysis of Survival

To further investigate independent prognostic factors in BCSD, the Fine–Gray multivariable regression model was established (Table 2). Results showed that patients in the pN0(i+) subgroup had higher BCSD rate (HR: 1.321, 95% CI = 1.109–1.575, *P* = 0.002) than patients in the pN0(i**−**) subgroup. Patients with young age, White and other races, highly differentiated (grade I), ER-positive tumors, PR-positive tumors, married state, radiotherapy, and no chemotherapy tended to have significantly lower BCSD than do the corresponding subgroups (*P* < 0.05). In addition, age, race, grade, marital status, and radiotherapy and chemotherapy status were also associated with non-BCSD (*P* < 0.05).

**TABLE 2:** Multivariable competing risk analysis in patients with pN0(i+) and pN0(i−) female breast cancer.

Characteristics	BCSD (N1 = 1,685, 20.97%)			Non-BCSD (N2 = 6,349, 79.03%)		
	Hazard ratio	95% CI	*P*-value	Hazard ratio	95% CI	*P*-value
Age (years, mean ± SD)	1.022	1.017–1.027	**<0.001**	1.097	1.094–1.100	**<0.001**
Stage N						
N0(i−)	1	–		1	–	
N0(i+)	1.321	1.109–1.575	**0.002**	1.009	0.906–1.125	0.86
Race						
Non-Hispanic White	1	–		1	–	
Non-Hispanic Black	1.568	1.357–1.813	**<0.001**	1.343	1.229–1.469	**<0.001**
Hispanic (all races)	1.262	1.073–1.486	**0.005**	0.966	0.873–1.068	0.50
Other races	0.800	0.649–0.986	**0.036**	0.670	0.594–0.756	**<0.001**
Laterality						
Left	1	–		1	–	
Right	1.031	0.9437–1.135	0.53	0.975	0.928–1.024	0.30
Grade						
1	1	–		1	–	
2	1.835	1.574–2.139	**<0.001**	1.066	1.008–1.128	**0.026**
3/4	3.288	2.768–3.906	**<0.001**	1.086	1.004–1.175	**0.040**
ER						
Positive	1	–		1	–	
Negative	1.509	1.279–1.782	**<0.001**	1.098	0.995–1.213	0.064
PR						
Positive	1	–		1	–	
Negative	1.311	1.128–1.525	**<0.001**	0.998	0.928–1.074	0.97
Marital status						
Married	1	–		1	–	
Unmarried/DSW	1.170	1.058–1.293	**0.002**	1.339	1.271–1.410	**<0.001**
Surgery						
BCS	1	–		1	–	
Mastectomy	1.147	0.987–1.334	0.074	0.964	0.898–1.035	0.31
Radiotherapy						
Yes	1	–		1	–	
No	1.220	1.060–1.404	**0.006**	1.350	1.268–1.438	**<0.001**
Chemotherapy						
Yes	1	–		1	–	
No	0.817	0.720–0.928	**0.002**	1.227	1.119–1.346	**<0.001**

*ER, estrogen receptor; PR, progesterone receptor; DSW, divorced and separated and widowed; BCS, breast-conserving surgery. Bold values means P < 0.05.*

### Chemotherapy, Radiotherapy, and Survival for Patients With pN0(i+)

**FIGURE 4:**
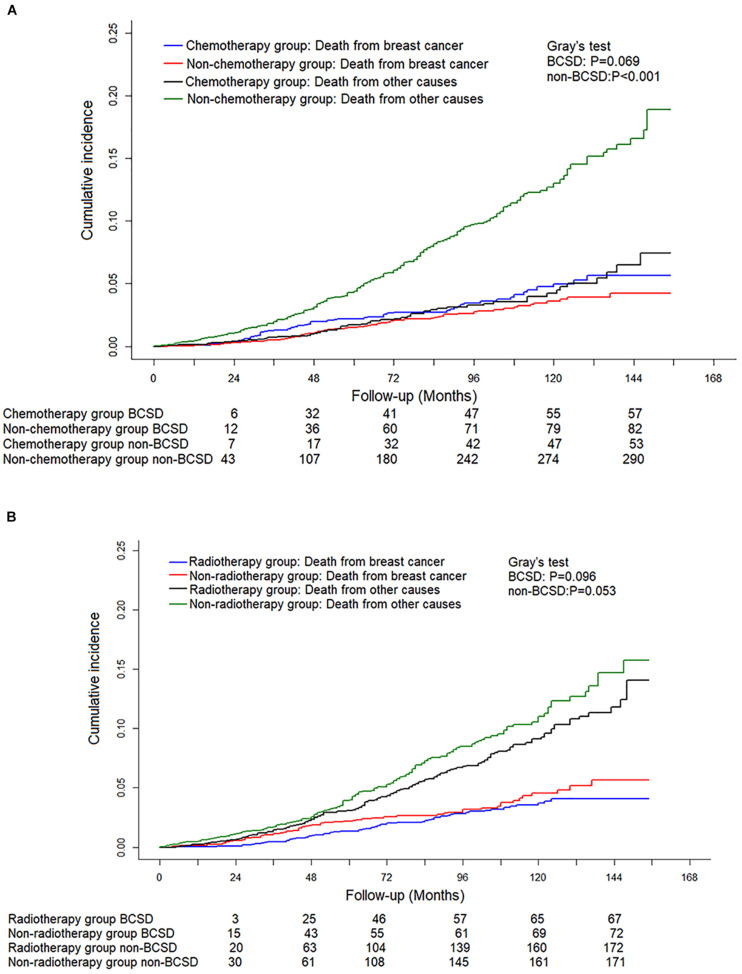
Competing risk analysis of the effect of different adjuvant treatment methods with surgery on the survival in pT0N0(i+) M0 female breast cancer patients. **(A)** Cumulative incidence of breast cancer-specific deaths (BCSDs) and non-breast cancer-specific deaths (non-BCSDs) in chemotherapy subgroup and non-chemotherapy subgroup. **(B)** Cumulative incidence of BCSDs and non-BCSDs in radiotherapy subgroup and non-radiotherapy subgroup.

Of the 5,967 pN0(i+) FBC patients included, 3,187 received radiotherapy, while 1,181 had chemotherapy. To further explore and identify the effect of treatment methods on the prognosis of patients staged with pT1N0(i+) M0, a competing risk model was performed. As shown in Figures 4A,B, no significant difference in cumulative BCSD was shown between chemotherapy and non-chemotherapy subgroups (Gray’s test, *P* = 0.069) or radiotherapy and non-radiotherapy subgroups (Gray’s test, *P* = 0.096).

## Discussion

In this retrospective study, based on the analysis of the large cohort of 94,599 patients in the SEER database from 2004 to 2015 and an integrated range of factors in the competing risk model, we confirmed that ITCs could significantly reduce BCSS for FBC patients. To our knowledge, this was the first and largest population-based study to assess the impact of ITCs on FBC patients by using the Fine–Gray multivariable regression model through analyzing survival variables, demographic characteristics, and pathological factors.

The H&E staining has been widely used to determine lymph node metastasis because it can easily identify single cells or cell clusters that were difficult to recognize in the past. However, the ITC was found in a considerable number of patients with H&E staining negative in the lymph nodes when more accurate IHC staining was performed. As a result, pN0(i**−**) and pN0(i+) have been added to the 6th edition of the AJCC guideline to specify whether ITC is detected. However, in the 8th edition of the AJCC guideline, the concepts of pN0(i**−**) and pN0 were merged and unified as pN0. This leads to a more consistent and precise definition of breast cancer occult metastases.

It has been recognized that the degree of lymph node spread is closely related to the outcome and risk of the tumor, which is also the reason why the degree of lymph node metastasis should be accurately defined. The volume of ITC is six times smaller than that of micrometastasis; therefore, some scientists call it “nano metastasis,” and they propose upgrading pN0(i+) to a new pathological category “pN1na” to emphasize the importance of ITC (17). The mechanism of ITC affecting prognosis may be that it can promote tumor progression and metastasis even as a nanoscale metastasis. The reason why the prognostic value of ITC is ignored may be related to the overall good prognosis of breast cancer and can also be interpreted as being covered by other confounding factors such as age, race, molecular type, and other clinicopathological features.

Breast cancer-specific survival is an objective, reliable, precise, and bias-free measurement for patients with breast carcinoma. In our study, after Kaplan–Meier curve analysis, patients in the pN0(i+) group had poorer BCSS than patients in the pN0(i**−**) group. However, the estimation bias resulting from BCSD and other competitive causes of death should not be neglected. The occurrence of competitive events hinders the possibility of events of interest (18, 19) and might be a competing risk affecting BCSS and preclude the occurrence of the primary event of BCSD. To mitigate the estimation bias and to further investigate the influence of ITCs on BCSD or other causes of death, a multivariable competing risk analysis was used. After multivariable competing risk analysis, the pN0(i+) group had a higher cumulative BCSD rate than the pN0(i**−**) group, and ITC was shown to be an independent prognostic factor.

In the National Surgical Adjuvant Breast and Bowel Project (NSABP) trial B-32, the effect of occult metastases on survival in node-negative breast cancer was studied through phase 3 clinical trials (4). They performed IHC in 3,887 breast cancer patients with node-negative breast cancer and found that isolated tumor-cell clusters were detected in 11.1% of patients. After a median follow-up period of 95.3 months, there was a significant difference in OS and BCSD between patients with ITC clusters and those without metastasis, and the HRs were 1.37 (95% CI = 1.03–1.81) and 1.38 (95% CI = 1.02–1.87), respectively. In our study, we found that there was no significant difference between the pN0(i+) and pN0(i**−**) groups in OS, but through the adjustment of the competing risk model, the pN0(i+) group had a higher BCSD rate (HR: 1.321, 95% CI = 1.109–1.575) than the pN0(i**−**) group. The reasons for the different results may be related to the number of population, follow-up time, population selection, or control of confounding factors. However, both the NSABP trial B-32 and our research show that it is necessary to test IHC in node-negative breast cancer patients, which determines the prognosis of patients and may affect the choice of treatment.

Traditionally, the selection of postoperative treatment after breast cancer depends on the pathological characteristics of the tumor, such as tumor size, lymph node status, histological classification, and molecular typing. It is not clear whether chemotherapy or radiotherapy is necessary for T1N0M0 FBC patients with ITCs in axillary lymph nodes. In our study, no significant difference in cumulative BCSD was shown between the chemotherapy and non-chemotherapy subgroups or radiotherapy and non-radiotherapy subgroups. These results were consistent with the previous reports (20). The underlining reason can be explained by ITCs that may have a low potential for malignancy; therefore, there is no extra survival benefit for additional radiotherapy or chemotherapy. Interestingly, we also found that chemotherapy in the Fine–Gray model analysis was a risk factor for patients staged with pT1N0M0 FBC. Randomized controlled clinical trials with long follow-up time are still needed to provide a high level of evidence on the advantage of chemotherapy for patients with pT1N0(i+) M0 FBC.

At present, gene detection is an accurate method to predict the prognosis of breast cancer. For example, 21 gene assay (Oncotype DX) is a prognostic marker for ER-positive and lymph node-negative breast cancer (21, 22). Seventy gene assay (MammaPrint) is closely related to the outcome of young patients with primary breast cancer of less than 5 cm (23, 24). The significance of 76 gene assay in lymph node-negative breast cancer has been confirmed (25, 26). However, gene detection is expensive, difficult to operate, and limited in accuracy; and the clinical application value needs to be further discussed. However, as traditional clinicopathological information, ITC detection is convenient, fast, cheap and has a significant correlation with prognosis. Therefore, in terms of predicting prognosis, ITC may be a better choice for lymph node-negative breast cancer.

In our study, we found that breast cancer is not the main cause of death in patients with pN0(i+) or pN0(i**−**), and non-BCSD accounts for the majority. During 10 years of follow-up, the non-BCSD rate of pN0(i+) and pN0(i**−**) was as high as 71.33 and 79.00%, respectively. A study on the causes of death for FBC patients has confirmed that the main cause of death was not breast cancer but heart disease 5 years after the diagnosis, and the proportion of BCSD will gradually decrease (27). Therefore, for patients with early-stage breast cancer, it is very important to pay close attention to other non-cancer diseases, especially cardiovascular health.

Inevitably, there are some limitations in this study that should not be ignored. First of all, as a retrospective study rather than a prospective cohort study, inherent selection biases and uncontrolled confounding factors cannot be avoided and may limit the external effects of this study. Secondly, we were unable to avoid the possibility that the observed risk reductions might exclude the influence of potential confounders, such as surgery methods of axillary lymph nodes, family history, comorbidities, endocrine therapy, targeted therapy against HER-2/neu-overexpression, and health status. These data greatly impacted the clinical decisions (28) and even breast cancer prognosis (29). Thirdly, as the data of human epidermal growth factor receptor 2 (HER-2)status were not available until 2010, therefore, we deleted the information about HER-2, which cannot clear the effect of HER-2 on survival.

## Conclusion

In conclusion, our study demonstrated that the presence of ITCs was independently related to the increase of the BCSD rate and could be identified as a reliable marker to predict the prognosis for patients with T1N0M0 FBC.

## Data Availability Statement

Publicly available datasets were analyzed in this study. This data can be found here: Surveillance, Epidemiology, and End Results (SEER) database (https://seer.cancer.gov/).

## Author Contributions

YL, WZ, and YR designed the experiments. YL, YQ, and HC collected and analyzed the data. YL, KL, and SP created the tables and figures and drafted the manuscript. YL and KL contributed reagents, materials, and analysis tools. YL helped with the statistical methods. CZ and JH supervised the completion of the study. All authors discussed the results, contributed to the final manuscript, approved the final draft, and decided to submit it for publication.

## Conflict of Interest

The authors declare that the research was conducted in the absence of any commercial or financial relationships that could be construed as a potential conflict of interest.

## Footnotes

1 https://seer.cancer.gov/seerstat/

2 https://www.r-project.org/
